# Liquid biopsy using circulating miRNAs for the identification of recurrence-associated biomarkers in glioblastoma CNS WHO grade 4

**DOI:** 10.3389/fonc.2026.1938611

**Published:** 2026-10-02

**Authors:** Razan Muhtadi, Christian D. Diehl, Nikolai Schmid, Gabriele Multhoff, Denise Bernhardt, Stephanie E. Combs, Lisa Hönikl, Sandro M. Krieg, Jens Gempt, Bernhard Meyer, Philipp Karschnia, Arthur Wagner, Vahe Barsegian, Monika Lindemann, Samantha Stewart, Sabrina Fischer, Patrick Ostheim, Stefan Friedrich Eder, Michael Abend

**Affiliations:** 1 Bundeswehr Institute of Radiobiology, Munich, Germany; 2 Graduate Center of Medicine and Health, Technical University of Munich, Munich, Germany; 3 Department of Radiation Oncology, TUM School of Medicine and Health, Munich, Germany; 4 Institute of Radiation Medicine (IRM), Helmholtz Zentrum München GmbH, German Research Center, Munich, Germany; 5 Deutsches Konsortium für Translational Krebsforschung (DKTK), Partner Site Munich, Munich, Germany; 6 Department of Neurosurgery, TUM School of Medicine, Klinikum Rechts der Isar, Munich, Germany; 7 Department of Neurosurgery, University Hospital Heidelberg, Heidelberg University, Heidelberg, Germany; 8 Institute of Nuclear Medicine, Helios Kliniken, Schwerin, Germany; 9 Institute for Transfusion Medicine, University Hospital, Essen, Germany

**Keywords:** gene expression, glioblastoma CNS WHO grade 4, liquid biopsy, magnetic resonance imaging (MRI), qRT-PCR

## Abstract

**Introduction:**

Glioblastoma CNS WHO Grade 4 (GBM) is associated with poor prognosis and high recurrence rates despite the current therapeutic approaches. Conventional imaging, in particular Magnetic Resonance Imaging (MRI), is primarily used during follow-up to detect tumor recurrence but has limited ability to distinguish recurrence from therapy-related changes, such as tissue necrosis and postoperative scar tissue. Liquid biopsy using circulating tumor-specific serological biomarkers, such as miRNAs, provides a gene expression signature for early identification of GBM recurrence. This pilot “proof-of-concept” study investigated the potential of circulating tumor-derived miRNA in whole blood samples for early detection of tumor recurrence.

**Methods:**

In a previous whole-transcriptome screening (NGS) study, whole-blood samples (n = 33) from seven patients and tumor biopsies (n = 4) were used to identify biomarkers. Corresponding to tumor surgery and recurrence, miRNA expression was examined in pre-surgical blood samples and tumor tissues. In whole blood, we investigated whether a pattern could be identified indicating post-surgery downregulation, consistent with reduced tumor burden, followed by a return to pre-surgery levels at recurrence. We identified 1–19 miRNA species per patient (a total of 72 miRNAs) showing this pattern. In this manuscript, we present the qRT-PCR validation results. The 45 most promising miRNAs identified by NGS study were selected and quantified using qRT-PCR. In addition, *miR-21* was included as a promising candidate reported in the literature. To address challenges in miRNA normalization, five normalization strategies and unnormalized raw Ct values were compared.

**Results:**

Two normalization approaches (median DCt normalization and unnormalized raw Ct values) showed no systematic bias. Depending on the normalization method, the agreement between NGS and qRT-PCR ranged from 23.6 to 27.9%. Although the concordance between NGS and qRT-PCR was low at the individual-patient level, qRT-PCR identified a common set of 24 miRNAs differentially regulated across patients. Notably, *miR-424-3p* and *miR-362-5p* were shared by five of seven patients, followed by *miR-1307-3p* and *miR-22-5p*, each shared by four patients. Interestingly, *miR-21* did not show the predefined recurrence-associated pattern in any patient.

**Discussion:**

Blood-based miRNA profiling may complement imaging in longitudinal monitoring of GBM patients, although clinical benefits have not been confirmed, and validation in a larger independent cohort is required.

## 1 Introduction

Glioblastoma (GBM), IDH wild-type, World Health Organization central nervous system (WHO CNS) grade 4, represents the most common and aggressive primary malignant brain tumor (1, 2). Despite the multimodal treatment consisting of maximal safe surgical resection followed by radiotherapy and concomitant and adjuvant temozolomide chemotherapy according to the Stupp protocol, patient outcomes remain poor (3). Median overall survival is approximately 12–15 months, and long-term survival beyond five years is uncommon (1, 4). Disease recurrence is nearly universal and remains the principal cause of treatment failure and mortality (5).

Accurate and early detection of tumor recurrence is a critical aspect of clinical management. The most commonly used imaging technique for diagnosis, treatment planning, and long-term monitoring is magnetic resonance imaging (MRI) (6, 7). Nevertheless, conventional MRI has well-recognized limitations in distinguishing true tumor progression from treatment-related changes, including radiation-induced necrosis, pseudoprogression, and post-operative scarring (8–10). Tissue biopsy remains the gold standard for diagnosis when imaging results are unclear. The necessity for supplementary, minimally invasive diagnostic techniques is highlighted by the fact that repeated neurosurgical interventions are invasive and involve significant risks, such as hemorrhage, cerebral edema, infarction, and neurological impairments (9, 11).

By analyzing tumor-specific serological biomarkers in body fluids, liquid biopsy has emerged as a promising approach for monitoring and tracking tumors (12). Liquid biopsies have several advantages, including the non-invasive ability to detect tumor molecular heterogeneity through comprehensive tissue profiling and their quick, easy acquisition. It can contain circulating tumor cells (CTCs), extracellular vesicles (EVs), and cell-free nucleic acids (cfDNA, cfRNA) (13). The cargo of EVs, including exosomal mRNA and miRNA, is already a promising marker for detecting tumors in certain types of cancer, such as pancreatic cancer and renal cell carcinoma (14).

The majority of liquid biopsy studies focus on isolating EVs from peripheral blood as an alternative to tumor biopsies. Nevertheless, this process is labor-intensive, yields low amounts of isolated RNA, and is difficult to implement in day-to-day routine clinical practice (15–17). In contrast, whole-blood transcriptomic profiling is a practical, clinically feasible alternative for identifying tumor-associated molecular signatures. As a proof-of-concept study, we examined the suitability of this less labor-intensive approach and whole-blood samples for liquid biopsy-based biomarker detection.

As a first step, we already performed whole-transcriptome sequencing (NGS) on whole-blood samples from seven patients (18). This analysis identified several candidate miRNA biomarkers whose expression levels decreased following tumor resection and subsequently returned toward baseline levels or even more at the time of recurrence. As a second step, based on the screening results, we aimed to validate the most promising miRNA biomarkers using customized TaqMan^®^ array cards (TACs). The results of this analysis are shown in this manuscript. Developing a liquid biopsy approach could enhance disease surveillance, allow earlier and individualized clinical decision-making for GBM patients, and supplement traditional imaging.

## 2 Materials and methods

### 2.1 Patient selection, inclusion criteria, and ethical approval

The same seven patients from the previous screening study with histologically confirmed Glioblastoma WHO CNS Grade 4 were enrolled in this validation study (18). All diagnoses were reviewed in accordance with the World Health Organization’s (WHO) revised classification of central nervous system tumors from 2021. As the WHO classification criteria were updated during the study, all patient diagnoses were retrospectively verified to ensure compliance with the revised 2021 criteria. The patients were treated at the Rechts der Isar clinic, Technical University of Munich (TUM), from 2019 to 2023. Inclusion criteria included patients aged ≥18 years undergoing adjuvant chemotherapy (Stupp scheme) and not taking immunosuppressive therapy. Exclusion criteria included long-term treatment with high-dose COX inhibitors, histone acetylation or DNA methylation affecting substances (e.g., valproic acid), and tricyclic antidepressants. Crucially, patients who suffered from a radiologically confirmed tumor recurrence during the follow-up period were included.

All patients received standard-of-care treatment in accordance with the Stupp protocol (19). In brief, patients underwent maximally safe surgical resection, followed by intensity-modulated radiotherapy (IMRT) with a total dose of 60 Gy in daily fractions of 2 Gy, delivered five days per week over six weeks. The accompanying chemotherapy consisted of a daily dose of temozolomide (TMZ) 75 mg/m²/day throughout the course of radiotherapy. Following completion of chemoradiotherapy, patients received six adjuvant cycles of TMZ (150–200 mg/m²/day on five consecutive days every 28 days). In addition, three patients received intraoperative radiotherapy (IORT) during resection of the primary tumor. All patients subsequently experienced local tumor recurrence, with a median time to recurrence of 158.3 (SD± 79.3) days after the first surgery.

Informed consent was obtained from all patients, and ethical approval for the study was obtained from the responsible Ethics Committee of the Technical University of Munich, Germany (approval number 572/16 S). All methods described in this manuscript (e.g., TaqMan^®^ qRT-PCR) were performed according to the relevant guidelines, regulations, and standard operating procedures implemented at the Bundeswehr Institute of Radiobiology in 2008 when the Institute became certified according to (DIN EN ISO 9001/2008), and were described in detail elsewhere (20, 21).

### 2.2 Blood samples collection and processing

Whole blood samples (2.5 mL) were collected from patients via venipuncture into PAXgene Blood RNA tubes (BD Diagnostics PreAnalytiX GmbH, Hombrechtikon, Switzerland) at predefined time points: pre-surgery, post-surgery, and pre/post- tumor recurrence (**Figure 1**, **Table 1**). Immediately after collection, the tubes were gently inverted ten times to ensure proper mixing with the stabilizing reagent, then stored overnight at room temperature to allow for complete RNA stabilization. The samples were then stored at −20 °C until further processing. All PAXgene tubes were transported under refrigerated conditions to the Bundeswehr Institute for Radiobiology for further processing.

**Figure 1:**
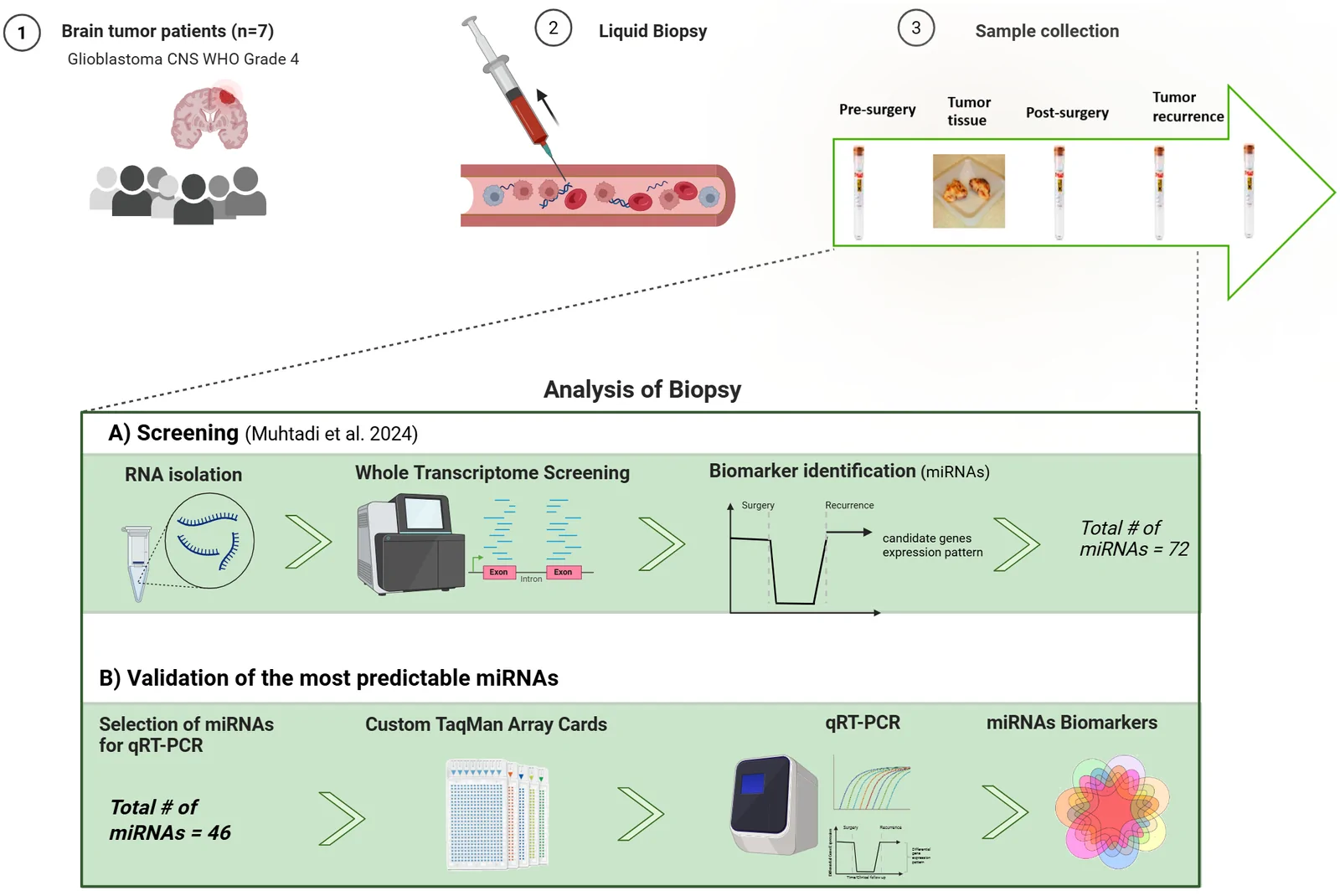
This figure shows the study workflow for identifying and validating circulating miRNA biomarkers associated with GBM recurrence (a simplified liquid biopsy approach). Peripheral whole-blood samples were collected from seven patients with glioblastoma CNS WHO grade 4 at defined clinical time points, including pre-surgery, post-surgery, and tumor recurrence. Tumor tissue was additionally included during the screening phase. **(A)** In the initial, already published screening phase, RNA was isolated and analyzed by whole-transcriptome NGS to identify miRNAs showing the predefined recurrence-associated expression pattern, characterized by downregulation after surgery and return toward pre-surgery levels at tumor recurrence. This screening approach identified 72 candidate miRNAs. **(B)** The current study focuses on the validation of the most predictive selected miRNAs using custom TaqMan array cards and qRT-PCR. In total, 46 miRNAs were examined to validate NGS-derived candidates and identify additional miRNAs showing the expected longitudinal expression pattern. GBM, glioblastoma; miRNA, microRNA; NGS, next-generation sequencing; qRT-PCR, quantitative reverse-transcription polymerase chain reaction. Created with Biorender.com.

**Table 1:** Patients’ characteristics, including sex and age.

Patient ID	Sex	Age (years)	Primary tumor tissue	Total # of blood samples	Days of blood sampling		
					Pre-surgery	Post-surgery	Pre-/post-recurrence
1	F	65	yes	4	1	5, 84	3
2	M	37	yes	4	1	6, 28	1
3	M	60	yes	6	1	6, 19, 29, 97	0
4	M	79	yes	3	1	7	0
5	F	45	no	5	5	28, 97, 184	-13
6	M	60	no	5	1	9, 27	0, 14
7	M	59	no	6	1	7, 17, 23	4, 17

Table 1 summarizes the characteristics of the seven examined patients, including sex and age. The table also provides an overview of the time and total number of blood samples taken from each patient. The blood samples were divided into three groups: pre-surgery, post-surgery, and pre/post-recurrence, the latter corresponding to the latest time point following surgery. The corresponding sampling time points in the table are given in days.

### 2.3 RNA extraction and quality control

RNA was extracted from whole-blood samples in PAXgene tubes. After thawing the PAXgene tubes, followed by washing and centrifugation steps, cells in the supernatant were lysed (Proteinase K), and a Lysis/Binding Solution from the mirVana kit™ (Life Technologies, Darmstadt, Germany) was added. Using the mirVana kit™ (Life Technologies), total RNA, including small RNA species, was isolated by combining phenol-chloroform RNA precipitation with further processing using a silica membrane. After several washing steps, DNA residues were digested on the membrane using the RNase-free DNase Set (Qiagen, Hilden, Germany). RNA was eluted in a collection tube and frozen at −20 °C.

The quality and quantity of isolated total RNA were determined spectrophotometrically using a NanoDrop (PeqLab Biotechnology, Erlangen, Germany). RNA integrity was assessed using both the 4200 TapeStation System and the 2100 Agilent Bioanalyzer (both Agilent Technologies, Santa Clara, CA, USA). DNA contamination was assessed by PCR using an actin primer pair (Sense: AGAGATGGCCACGGCTGCTT; antisense: ATTTGCGGTGGACGATGGAG). Only RNA samples with an A260/A280 nm ratio ≥ 2.0 (Nanodrop) and RNA integrity number (RIN) ≥ 7 for blood samples were utilized for the qRT-PCR validation step.

### 2.4 Screening phase for miRNAs

In a previous study, we conducted a whole-transcriptome screening using next-generation sequencing (NGS) of whole-blood samples and tumor tissues collected from the seven GBM CNS WHO grade 4 patients. miRNA candidates were selected using a predefined three-step filtering strategy: 1) miRNA candidates should be expressed in both tumor and blood samples prior to surgery, 2) at least a two-fold downregulation in gene expression following tumor resection. This filter was based on the assumption that surgical resection significantly reduces or completely removes the tumor mass, so we expected the expression levels of tumor-associated miRNAs in peripheral blood to decrease accordingly with a reduced or absent tumor burden, and 3) in case of tumor recurrence, an upregulation in gene expression to pre-surgery levels or even higher (18).

### 2.5 Selection criteria for miRNA candidates

To reduce the number of miRNA candidates for the validation phase, predefined selection criteria were applied. Priority was given to miRNAs that exhibited a consistent fold change (FC) of ≤0.6 across all postoperative time points. In addition, miRNAs with a FC ≤0.6 at any postoperative time point were considered for inclusion on an individual-patient basis. Furthermore, miRNAs previously associated with GBM and detected during the NGS screening phase were included, regardless of their fold change values. An exception was made for patient 2, as only a single miRNA was identified during the NGS analysis. Although this candidate did not meet the predefined selection criteria, it was included in the validation phase as no other candidates were available for this patient. The application of these criteria led to the selection of 45 miRNAs for validation from a total of 72 miRNAs identified during the screening phase (**Supplementary Table 1**). Furthermore, *hsa-miR-21-5p* was included as a potential biomarker, despite not being detected during the screening phase, given its well-documented association with GBM in the literature (22).

Three endogenous reference genes were used for data normalization due to the well-known challenges regarding normalization. The TaqMan^®^ Array Cards (TACs) contained *hsa-miR-103a-3p*, which is commonly used as a reference miRNA in GBM studies (22), and *hsa-miR-16-5p*, which is recommended by Thermo Fisher Scientific for TaqMan^®^ Advanced miRNA Assays. Additionally, an endogenous control not spotted on the TACs, *18S rRNA* (Hs99999901_s1), was used. Regarding the detection of *18S rRNA*, aliquots of RNA were reverse transcribed with the High-Capacity cDNA Reverse Transcription Kit (Applied Biosystems™ by Thermo Fisher Scientific, Germany), then diluted to 0.01ng per reaction and mixed with the TaqMan^®^ Universal PCR Master (Applied Biosystems™ by Thermo Fisher Scientific, Germany) and examined using conventional 96-well plates (Thermo Fisher Scientific, Germany).

### 2.6 Validation of the selected miRNAs using TaqMan^®^ assay cards

Using customized TaqMan^®^ array cards (Life Technologies), we employed a validation approach to verify the expression of 46 miRNAs, which were spotted in duplicate. Total RNA (10 ng) from whole blood samples was subjected to reverse transcription using TaqMan^®^ Advanced miRNA cDNA Synthesis Kit (Applied Biosystems™, Life Technologies, Darmstadt, Germany). Poly(A) tailing was performed on the RNA, followed by adapter ligation. The reverse transcription was then carried out, and finally, the miR-amplification step. The cDNA was diluted 1:10 with 0.1x TE buffer, then mixed with TaqMan^®^ Fast Advanced Master Mix (Applied Biosystems™, Life Technologies) and RNase-free water in a 1:2:1 ratio. Then, 100 µL of the mixture was pipetted into the eight fill ports of the TACs. The TACs were centrifuged twice at 1200 rpm for 1 minute (Multifuge 3S-R, Heraeus, Hanau, Germany), sealed, and then processed using QuantStudio™ 12K OA Real-Time PCR System (Applied Biosystems, Waltham, MA, USA). The qRT-PCR was run under the following cycling conditions: 92 °C (10 minutes), and 40 cycles of 95 °C (1 second) and 60 °C (20 seconds). The relative threshold algorithm was used, and a Ct value of 32 was identified as the cutoff point where the linear dynamic range of the method ends. To address the issue of miRNA normalization (23), *hsa- miR-103a-3p*, *hsa-miR-16-5p*, and *18S rRNA* were used as reference genes for data normalization, as well as the median Ct (referred to as the median Ct method), which was calculated from all 46 detected miRNA species. Additionally, the delta median from raw Ct values (referred to as the median ΔCt method) of two samples (the pre-OP sample was used as the reference sample) was calculated. The raw Ct values without employing further normalization approaches were examined as well. To calculate differential gene expression (DGE), pre-surgery blood samples were used as the baseline reference, employing the 2^-ΔΔCt method.

## 3 Results

### 3.1 Validation of post-transcriptional (miRNAs) candidates using TaqMan^®^ assay cards

A total of 45 miRNAs identified in seven patients were selected for validation using TaqMan^®^ assay cards (**Supplementary Table 1**). In addition, *miR-21* was included because previous literature suggests its potential relevance as a biomarker in GBM. All 46 miRNAs exhibited amplification curves within the measured linear dynamic range and were therefore included in the subsequent analysis.

### 3.2 Assessment of miRNA normalization approaches

The normalization approaches included three endogenous reference markers, namely *miR-103a-3p*, *miR-16-5p*, and *18S rRNA*, as well as median normalization and the median-delta raw Ct values across two samples (median ΔCt, using the pre-surgery raw Ct-values as the reference). Patient 1 is shown in **Figure 2** as a representative example.

**Figure 2:**
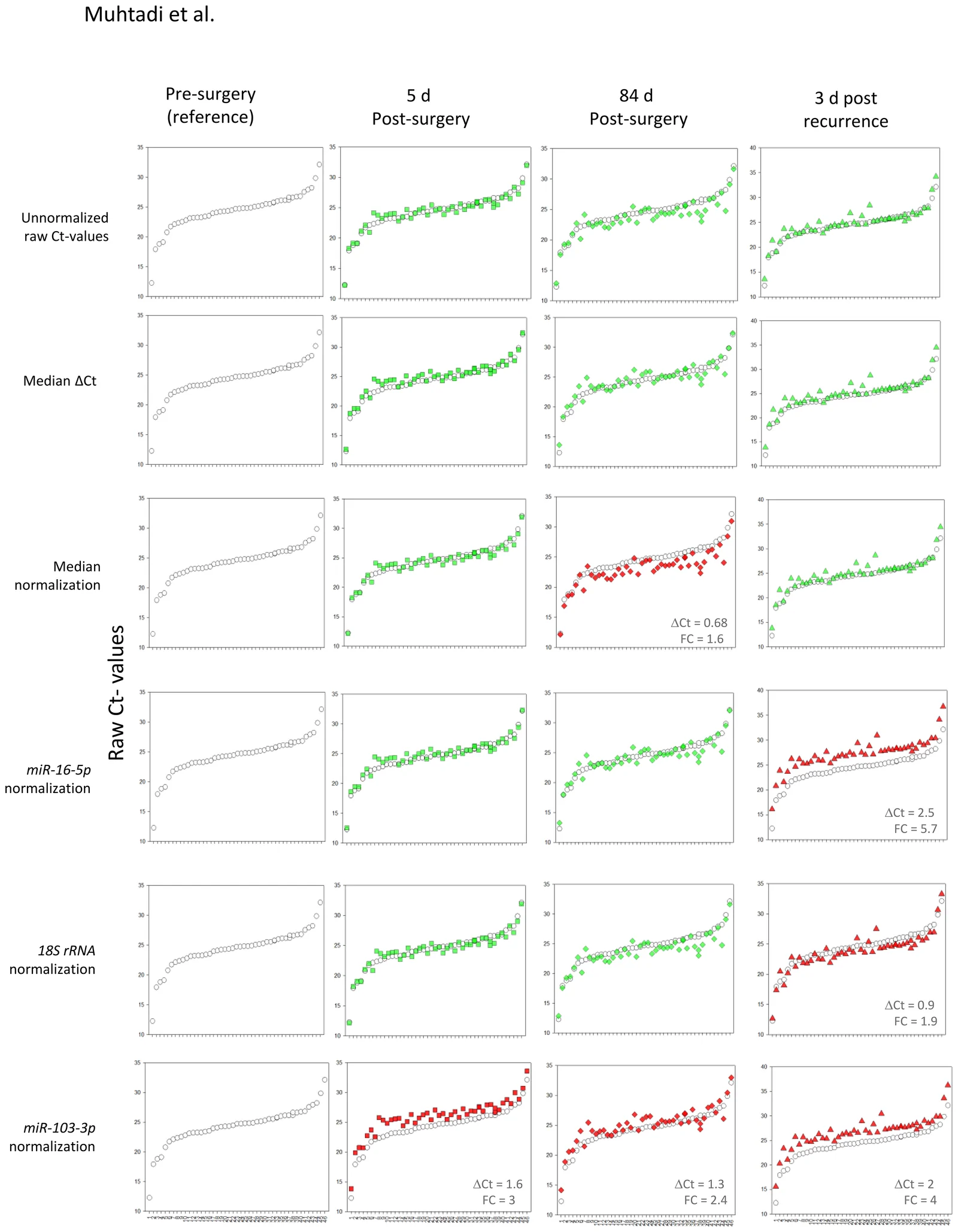
As an example, this figure shows the different normalization methods and their effects on the threshold-cycle (Ct) distribution for patient 1 across time points: pre-surgery reference sample (left column), 5- and 84-days post-surgery (middle columns), and 3 days post-recurrence (right column). Different normalization approaches are ranked (from the most plausible to the highly biased) in rows as follows: unnormalized raw Ct values, median ΔCt, Median, *miR-16-5p*, *18S rRNA*, and *miR-103-3p*. The bias results are shown in red and the plausible results in green. Ct-values within the graphs are ordered from lowest to highest. Systematic upward and downward shifts observed in 70% and more of the examined miRNAs were expressed as a mean fold-change relative to the pre-surgery reference.

To assess the plausibility of the individual normalization strategies, the Ct distributions following normalization across all patients and time points were compared with the corresponding pre-surgery reference values (**Figure 2**, **Table 2**). A normalization approach was considered biased if ≥70% of the 46 analyzed miRNAs showed a systematic upward or downward shift compared to the pre-surgery sample. Such a comprehensive shift was considered biologically implausible, as NGS screening had identified only a limited number of differentially expressed miRNA candidates per patient.

**Table 2:** This table presents the results of a plausibility check for five normalization approaches, along with unnormalized raw Ct-values.

Normalization strategies		Agreement between the pre-surgery reference raw Ct-values and the samples raw Ct-values after normalization					Total # samples/ patient	Agreement		Normalization strategies		Agreement between the pre-surgery reference raw Ct-values and the samples raw Ct-values after normalization					Total # samples/ patient	Agreement	
	Patient ID	# 1	# 2	# 3	# 4	# 5		Absolute (Total)	Percent (%)		Patient ID	# 1	# 2	# 3	# 4	# 5		Absolute (total)	Percent (%)
*miR-103-3p*	4	bias	bias				2			*18S rRNA*	4	bias	bias				2		
	1	bias	bias	bias			3				1	ok	bias	bias			3		
	2	bias	ok	ok			3				2	bias	ok	ok			3		
	5	ok	bias	bias	bias		4				5	bias	ok	bias	bias		4		
	6	bias	bias	ok	bias		4				6	bias	ok	ok	bias		4		
	3	ok	bias	ok	ok	ok	5				3	ok	ok	ok	ok	ok	5		
	7	ok	bias	bias	bias	ok	5	10	38.5		7	bias	ok	bias	ok	bias	5	13	50.0
*miR-16-5p*	4	bias	bias				2			Median ΔCt	4	ok	ok				2		
	1	ok	ok	bias			3				1	ok	ok	ok			3		
	2	ok	bias	ok			3				2	ok	ok	ok			3		
	5	ok	bias	ok	bias		4				5	ok	ok	ok	ok		4		
	6	bias	bias	ok	bias		4				6	ok	ok	ok	ok		4		
	3	ok	bias	ok	ok	bias	5				3	ok	ok	ok	ok	ok	5		
	7	ok	bias	bias	ok	ok	5	13	50.0		7	ok	ok	ok	ok	ok	5	26	100
Median	4	bias	ok				2			Unnormalized Raw Ct values	4	ok	ok				2		
	1	ok	bias	ok			3				1	ok	ok	ok			3		
	2	ok	bias	ok			3				2	ok	ok	ok			3		
	5	ok	bias	ok	ok		4				5	ok	ok	ok	ok		4		
	6	bias	bias	ok	bias		4				6	ok	ok	ok	ok		4		
	3	ok	bias	ok	ok	ok	5				3	ok	ok	ok	ok	ok	5		
	7	ok	ok	bias	bias	ok	5	16	61.5		7	ok	ok	ok	ok	ok	5	26	100

We examined whether there was a systematic shift in normalized Ct values relative to the pre-surgery sample for each patient. Patients are ordered by increasing sample count (given as #1-5), and the total number of samples is indicated for each patient in a separate column. For each patient and sample, the comparison is classified as ok (green, indicating plausible agreement with the pre-surgery reference) or bias (red, indicating a systematic shift from the pre-surgery reference). The overall agreement is summarized in absolute counts and percentages over all patients and separately for each normalization method.

Unnormalized raw Ct values and median ΔCt normalization showed the most consistent distributions, closely aligning with the pre-surgery reference in patient 1 (**Figure 2**) and across all patients (**Table 2**). In contrast, normalization using *miR-103a-3p* produced a systematic shift in all samples from patient 1, with a maximum ΔCt of 2, corresponding to a 4-fold change. Normalization with *miR-16-5p*, *18S rRNA*, and the median normalization resulted in a systematic shift in one sample each, with maximum ΔCt values of 2.5, 0.9, and 0.7, corresponding to fold changes of 5.7, 1.9, and 1.6, respectively (**Figure 2**).

Across all patients, *miR-103a-3p* showed the lowest agreement with the raw Ct reference value, with a concordance of 38.5% and a bias of 61.5% (**Table 2**). Normalization using *miR-16-5p* and *18S rRNA* each resulted in a deviation of 50%, whereas the median normalization yielded a deviation of 38.5%. In contrast, both the unnormalized raw Ct values and the median ΔCt normalization showed no detectable systematic deviation according to the predefined plausibility criteria (**Table 2**).

### 3.3 Analysis of the complete candidate miRNA panel using qRT-PCR

All 46 candidate miRNAs selected during the NGS discovery phase were subsequently analyzed by qRT-PCR in all seven patients, regardless of whether a specific miRNA had met the NGS filter criteria in any individual patient. Accordingly, a standardized panel of candidate genes was analyzed in all patients as part of the qRT-PCR analysis, whereas the NGS discovery analysis generated patient-specific sets of candidate genes.

The number of miRNAs derived from NGS data selected for qRT-PCR validation varied among patients, ranging from 1 to 14. Accordingly, the number of miRNA candidates that were not selected as part of the patient-specific NGS filtering process but were nevertheless included in the standardized qRT-PCR panel (revealing a consistent post-surgery decrease and a return to pre-surgery levels) was examined with the TACs for the GE pattern ranged from 31 to 44 (**Table 3**, first three columns to the left).

**Table 3:** This table illustrates the results of the validation step using quantitative real-time PCR (qRT-PCR) for gene expression patterns identified during the screening phase using Next-Generation Sequencing (NGS) across patient samples.

			Unnormalized raw Ct values				Median ΔCt			
Patient	# NGS candidate miRNAs selcted per patient	# of non-candidate (remaining) miRNAs	NGS miRNAs showing the expected pattern via qRT-PCR		Non-NGS candidates miRNAs showing the expected pattern via qRT-PCR		NGS miRNAs showing the expected pattern via qRT-PCR		Non-NGS candidate miRNAs showing the expected pattern via qRT-PCR	
			#	%	#	%	#	%	#	%
1	14	31	3	21.4	2	6.5	4	28.6	6	19.4
2	1	44	0	0.0	3	6.8	0	0.0	3	6.8
3	3	42	2	66.7	11	26.2	2	66.7	6	14.3
4	9	36	1	11.1	2	5.6	3	33.3	4	11.1
5	5	40	1	20.0	4	10.0	1	20.0	2	5.0
6	6	39	2	33.3	5	12.8	1	16.7	4	10.3
7	7	38	3	42.9	18	47.4	0	0.0	10	26.3
				27.9		16.5		23.6		13.3

The left section shows the number of NGS candidate genes selected for each patient, and the remaining number (non-NGS candidates) for each patient. Both numbers combined sum up to 45 miRNAs, since all miRNAs were examined in all patients and samples. The middle section shows the absolute counts and percentages of NGS candidate genes and the remaining (non-NGS) candidates that exhibit the expected gene expression pattern using unnormalized raw Ct values, and the right section shows the results using median ΔCt for normalization. These two most promising approaches among the five examined normalization procedures are shown.

Using unnormalized raw Ct values, 27.9% of the NGS miRNA candidates were successfully validated by qRT-PCR. Furthermore, 16.5% of the miRNA candidates that had not fulfilled the patient-specific NGS selection criteria exhibited the predefined longitudinal gene expression pattern when analyzed by qRT-PCR (**Table 3**, middle part). Median ΔCt normalization yielded a validation rate of 23.6% for NGS miRNA candidates and identified the expected pattern in 13.3% of the remaining miRNAs (**Table 3**, last remaining columns to the right).

### 3.4 Consistency between NGS and qRT-PCR results

**Table 4** compares the normalization approaches applied to the NGS data from patient 1. In accordance with the predefined plausibility criteria, both the unnormalized raw Ct values and the normalization based on the median of ΔCt identified miRNAs with expression patterns that were consistent with the NGS results.

**Table 4:** Presents differential gene expression (DGE) results of patient 1 relative to pre-surgery samples during the clinical follow-up, as indicated (pre-surgery, post-surgery, and tumor recurrence time points).

					QRT-PCR data normalization procedures using:																								
	NGS data				*miR-103a-3p*				*18S rRNA*				*miR-16-5p*					Median				Median delta raw Ct of two samples				Unnormalized raw Ct values			
Patient# 1	Pre-surgery	Post-surgery		Post-recurrence	Pre-surgery	Post-surgery		Post-recurrence	Pre-surgery	Post-surgery		Post-recurrence	Pre-surgery		Post-surgery		Post-recurrence	Pre-surgery	Post-surgery		Post-recurrence	Pre-surgery	Post-surgery		Post-recurrence	Pre-surgery	Post-surgery		Post-recurrence
*miRNAs ID*	*1d*	*5d*	*84d*	*3d*	*1d*	*5d*	*84d*	*3d*	*1d*	*5d*	*84d*	*3d*	*1d*		*5d*	*84d*	*3d*	*1d*	*5d*	*84d*	*3d*	*1d*	*5d*	*84d*	*3d*	*1d*	*5d*	*84d*	*3d*
*hsa-let-7b-3p*	1	0.78	0.26	1.11	1.00	4.24	6.12	3.89	1.00	1.30	2.46	0.51	1.00		1.74	3.29	5.56	1.00	1.33	1.53	1.12	1.00	1.39	1.46	0.95	1.00	1.39	2.46	0.95
*hsa-miR-424-* *3p*	1	1.02	0.12	1.09	1.00	2.76	2.62	3.90	1.00	0.84	1.05	0.51	1.00		1.13	1.41	5.57	1.00	0.87	0.66	1.12	1.00	0.95	0.66	1.00	1.00	0.83	1.16	0.96
*hsa-miR-574-3p*	1	0.47	0.86	1.23	1.00	1.94	3.47	4.12	1.00	0.59	1.40	0.54	1.00		0.79	1.87	5.89	1.00	0.61	0.87	1.19	1.00	0.63	0.83	1.01	1.00	0.63	1.39	1.01
*hsa-miR-625-3p*	1	0.67	0.28	1.84	1.00	1.56	2.21	3.94	1.00	0.48	0.89	0.52	1.00		0.64	1.19	5.62	1.00	0.49	0.55	1.13	1.00	0.51	0.53	0.97	1.00	0.51	0.89	0.97
*hsa-miR-664a-3p*	1	0.57	0.13	1.01	1.00	1.85	3.24	2.00	1.00	0.56	1.30	0.26	1.00		0.76	1.74	2.86	1.00	0.58	0.81	0.58	1.00	0.60	0.77	0.49		0.60	1.30	0.49
*hsa-miR-1307-3p*	1	0.76	0.29	1.36	1.00	2.41	2.34	5.26	1.00	0.74	0.94	0.69	1.00		0.99	1.26	7.50	1.00	0.76	0.59	1.51	1.00	0.78	0.56	1.28	1.00	0.78	0.94	1.28
*hsa-miR-3909*	1	1.43	0.19	1.31	1.00	6.21	8.29	6.59	1.00	1.90	3.33	0.86	1.00		2.55	4.47	9.41	1.00	1.95	2.08	1.90	1.00	2.04	1.99	1.61	1.00	2.04	3.34	1.61
*hsa-miR-4732-3p*	1	0.89	0.4	1.12	1.00	1.77	5.72	3.71	1.00	0.54	2.30	0.49	1.00		0.73	3.08	5.29	1.00	0.56	1.43	1.07	1.00	0.58	1.37	0.91	1.00	0.58	2.30	0.91
*hsa-miR-5010-3p*	1	0.54	0.38	1.1	1.00	2.30	4.82	3.40	1.00	0.70	1.94	0.45	1.00		0.94	2.60	4.85	1.00	0.72	1.21	0.98	1.00	0.75	1.16	0.84	1.00	0.75	1.95	0.84
*hsa-miR-204-* *5p*	1	0.86	0	1.15	u.d.	u.d.	u.d.	u.d.	u.d.	u.d.	u.d.	u.d.	u.d.		u.d.	u.d.	u.d.	u.d.	u.d.	u.d.	u.d.	u.d.	u.d.	u.d.	u.d.	u.d.	u.d.	u.d.	u.d.
*hsa-miR-130b-5p*	1	1.04	0.29	2.12	1.00	2.68	7.69	4.36	1.00	0.82	3.09	0.57	1.00		1.10	4.14	6.22	1.00	0.84	1.92	1.25	1.00	0.88	1.84	1.06	1.00	0.88	3.10	1.06
*hsa-miR-1271-5p*	1	1.21	0.46	1.45	1.00	2.51	u.d.	5.00	1.00	0.77	u.d.	0.66	1.00		1.03	u.d.	7.14	1.00	0.79	u.d.	1.44	1.00	0.82	u.d.	1.22	1.00	0.82	u.d.	1.22
*hsa-miR-454-5p*	1	1.23	0.16	2.82	1.00	2.19	4.42	3.95	1.00	0.67	1.78	0.52	1.00		0.90	2.38	5.64	1.00	0.69	1.11	1.14	1.00	0.72	1.05	0.97	1.00	0.72	1.77	0.97
*hsa-miR-1306-5p*	1	0.41	0.11	2.85	1.00	1.31	2.17	3.48	1.00	0.40	0.87	0.46	1.00		0.54	1.17	4.97	1.00	0.41	0.54	1.00	1.00	0.43	0.52	0.85	1.00	0.43	0.88	0.85
*hsa-miR-1249-3p*					1.00	1.37	2.24	6.86	1.00	0.42	0.90	0.90	1.00		0.56	1.21	9.80	1.00	0.43	0.56	1.97	1.00	0.45	0.54	1.68	1.00	0.45	0.90	1.68
*hsa-miR-19a-3p*					1.00	2.37	1.27	1.04	1.00	0.72	0.51	0.14	1.00		0.97	0.69	1.49	1.00	0.74	0.32	0.30	1.00	0.77	0.30	0.26	1.00	0.77	0.51	0.26
*hsa-miR-378a-3p*					1.00	2.69	2.22	5.01	1.00	0.82	0.89	0.66	1.00		1.10	1.19	7.15	1.00	0.85	0.56	1.44	1.00	0.88	0.53	1.23	1.00	0.88	0.90	1.23
*hsa-miR-4286*					1.00	2.41	2.13	3.33	1.00	0.74	0.86	0.44	1.00		0.99	1.15	4.75	1.00	0.76	0.53	0.96	1.00	0.78	0.51	0.81	1.00	0.78	0.85	0.81
*hsa-miR-491-5p*					1.00	3.34	2.71	3.45	1.00	1.02	1.09	0.45	1.00		1.37	1.46	4.93	1.00	1.05	0.68	0.99	1.00	1.09	0.65	0.85	1.00	1.09	1.09	0.85
*hsa-miR-362-5p*					1.00	2.98	2.54	3.82	1.00	0.91	1.02	0.50	1.00		1.22	1.37	5.46	1.00	0.94	0.64	1.10	1.00	0.98	0.61	0.94	1.00	0.98	1.02	0.94
*hsa-miR-181d-5p*					1.00	2.27	2.88	4.36	1.00	0.69	1.16	0.57	1.00		0.93	1.55	6.23	1.00	0.71	0.72	1.26	1.00	0.74	0.69	1.07	1.00	0.74	1.16	1.07
*hsa-miR-16-2-3p*					1.00	0.88	0.72	1.68	1.00	0.27	0.29	0.22	1.00		0.36	0.39	2.40	1.00	0.28	0.18	0.48	1.00	0.29	0.17	0.41	1.00	0.29	0.29	0.41
*hsa-miR-210-3p*					1.00	1.94	1.11	4.32	1.00	0.59	0.45	0.57	1.00		0.80	0.60	6.17	1.00	0.61	0.28	1.24	1.00	0.64	0.26	1.06	1.00	0.64	0.44	1.06
*hsa-miR-185-5p*					1.00	3.05	1.95	4.27	1.00	0.93	0.78	0.56	1.00		1.25	1.05	6.10	1.00	0.96	0.49	1.23	1.00	1.00	0.47	1.04	1.00	1.00	0.78	1.04
*hsa-miR-484*					1.00	2.38	3.21	3.22	1.00	0.73	1.29	0.42	1.00		0.98	1.73	4.60	1.00	0.75	0.80	0.93	1.00	0.78	0.77	0.79	1.00	0.78	1.29	0.79
*hsa-miR-532-5p*					1.00	2.85	1.87	3.48	1.00	0.87	0.75	0.46	1.00		1.17	1.01	4.96	1.00	0.90	0.47	1.00	1.00	0.74	0.88	1.06	1.00	0.74	1.48	1.06
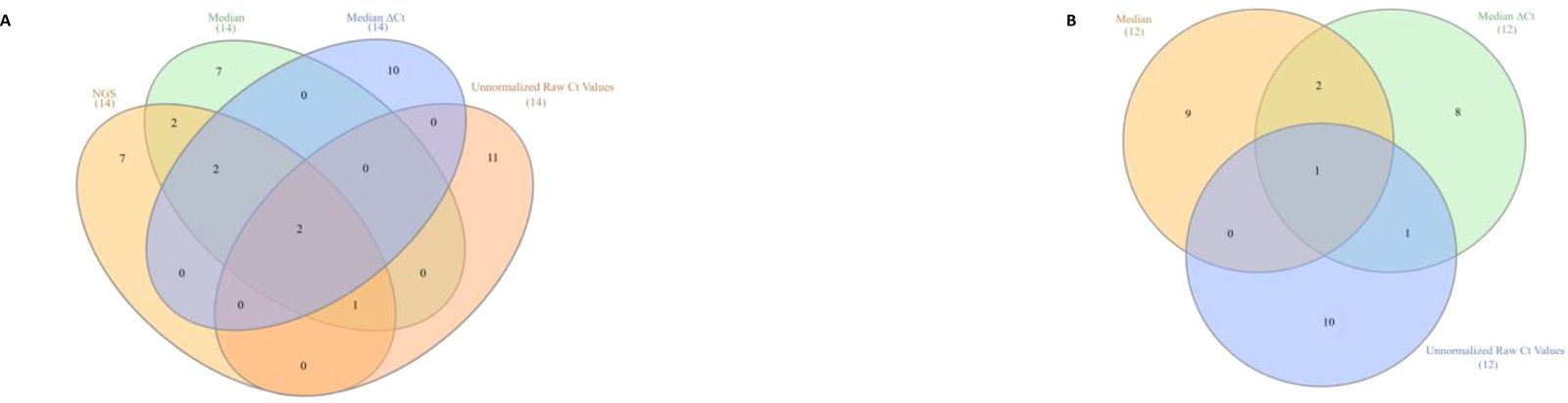																													

The 14 miRNA species identified in the NGS screening phase within a previous study are shown on the left. The DGE of qRT-PCR data of our current study is shown to the right, separately for each normalization method, as indicated in the subheaders. Cells highlighted in green indicate miRNAs that reveal the expected DGE pattern. The left Venn diagram below the table shows the overlap across methods, including NGS candidates from the screening phase and the different most promising qRT-PCR normalization methods as outlined. The right Venn diagram below in **Table 4** shows the overlap between qRT-PCR data for the most promising normalization strategies (as outlined) for the remaining genes that were not selected as part of the patient-specific NGS filtering process, but showed the defined gene expression pattern. d, day; u.d., undefined values.

Interestingly, the overlap analysis showed that *miR-574-3p* and *miR-625-3p* were detected among the selected NGS candidate miRNAs as well as in the qRT-PCR data after normalization using the “median,” “median ΔCt,” and “unnormalized raw Ct values” approaches. This overlap is illustrated in Venn diagram A in **Table 4**.

Furthermore, qRT-PCR identified miRNAs that exhibited the predefined expression pattern even though they were not selected by the patient-specific NGS filtering process. Examples include *miR-1249-3p* and *miR-210-3p*, as shown in **Table 3**, the lower part of **Table 4**, and summarized in Venn diagram B in **Table 4**.

### 3.5 Representative longitudinal miRNA DGE patterns

**Figure 3A** shows representative examples of the expected longitudinal miRNA expression pattern in patient 4. This Figure illustrates the agreement between the longitudinal expression patterns identified by NGS and those identified by qRT-PCR. These miRNAs were first identified using NGS and subsequently validated by qRT-PCR. For *miR-501-3p*, the analysis using unnormalized raw Ct values revealed a non-significant positive linear correlation with the NGS pattern (R² = 0.83, p = 0.27, **Figure 3A**, upper panel). Similarly, *miR-185-5p* showed a non-significant linear correlation (R² = 0.98, p = 0.096) after median ΔCt normalization. The expected expression pattern was also observed for miRNAs that were not selected by the patient-specific NGS filtering process, as shown for patient 4 in **Figure 3B**.

**Figure 3:**
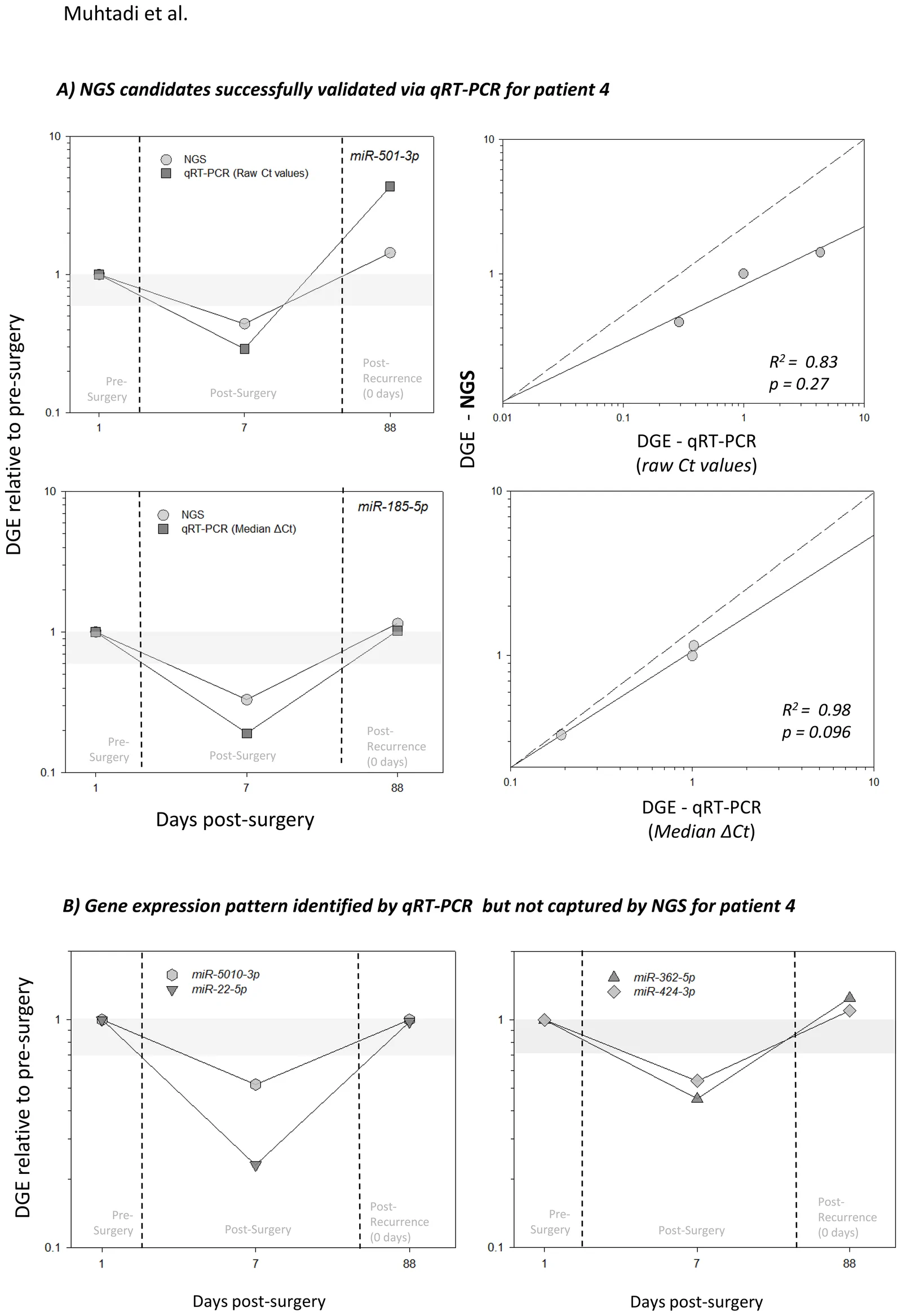
The upper part **(A)** of the figure shows examples of NGS candidates successfully validated by qRT-PCR for patient 4. In panel **(A)**, the left graph shows differential gene expression (DGE) over time for miR-501-3p and miR-185-5p. A correlation of DGE between the two methods (next-generation sequencing (NGS) and qRT-PCR) using either unnormalized raw Ct values or the median ΔCt normalization method is shown to the right. Phrases in grey (pre-surgery, post-surgery, and post-recurrence) refer to phases of the clinical follow-up for each patient. Deviations of DGE from expected values (referenced as a dotted line with y=x) indicate lower DGE detection when using qRT-PCR compared to NGS. Additional small RNAs that were not selected as part of the patient-specific NGS filtering process and that show the defined gene expression pattern using qRT-PCR are presented in **(B)** for the same patient.

### 3.6 Interpatient overlap of qRT-PCR validated miRNAs

After applying the predefined three-stage filtering strategy, the resulting miRNA candidates were compared across patients using an UpSet plot to visualize common and patient-specific candidates. The UpSet plot revealed considerable heterogeneity among the patients, including seven miRNAs detected exclusively in patient 7, as well as several overlaps involving only one to three miRNAs. Despite this heterogeneity, qRT-PCR identified a small group of miRNAs that were detected repeatedly across multiple patients. The most common candidates were *miR-424-3p* and *miR-362-5p*, each of which was present in five of seven patients. *miR-1307-3p* and *miR-22-5p* were each present in four patients. Another group of miRNAs was present in three patients, including *miR-1249-3p*, *miR-185-5p*, *miR-210-3p*, *miR-30e-5p*, *miR-378a-3p*, *miR-532-3p*, and *miR-625-3p*. In addition, 10 miRNAs were shared in two patients each, as shown in **Figure 4**. Overall, these results suggest that while NGS screening showed only minor overlap in differentially expressed miRNAs among patients, qRT-PCR analysis identified several recurring miRNA candidates across the entire cohort.

**Figure 4:**
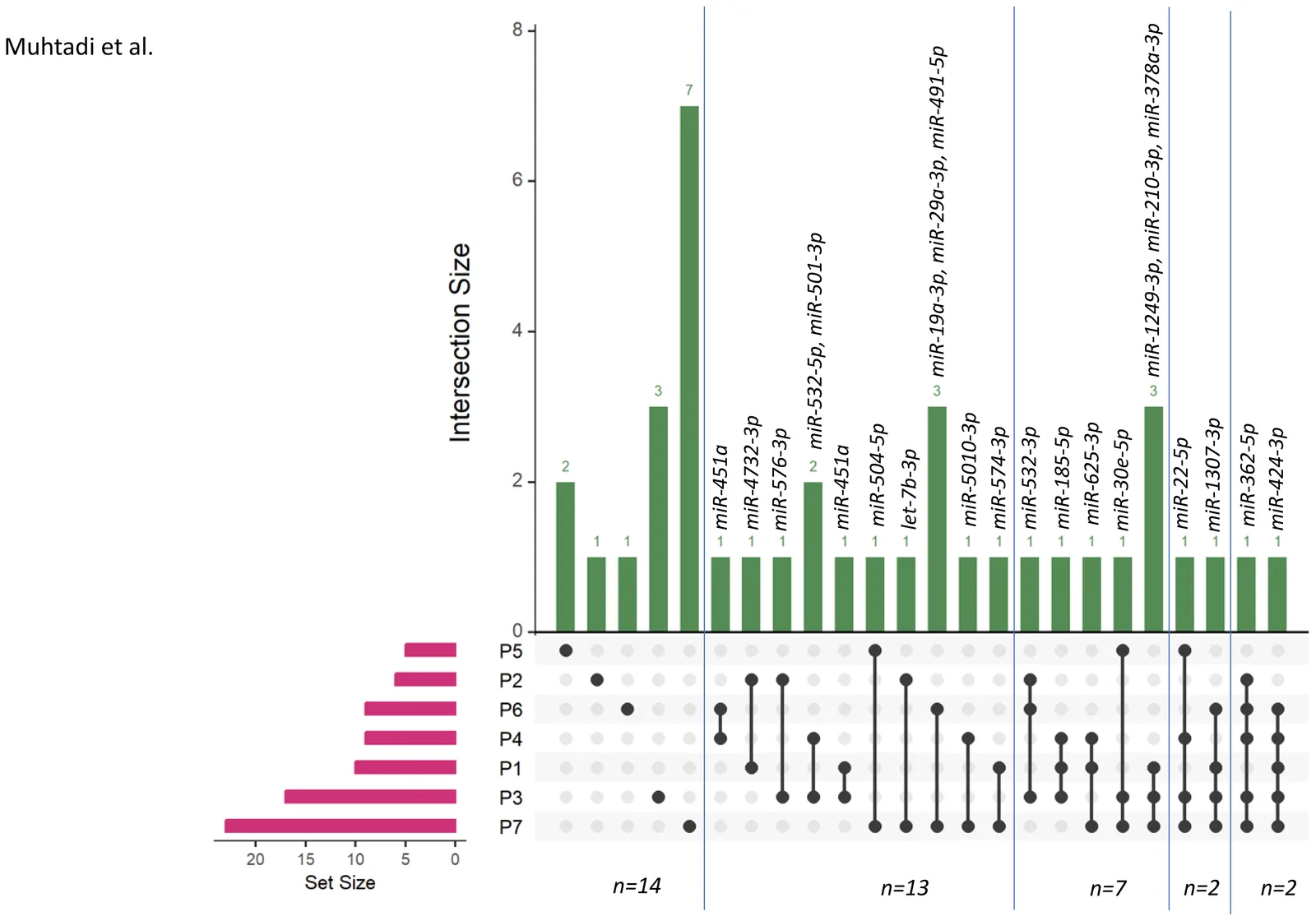
The UpSet plot provides a descriptive visualization of candidate miRNA overlaps across patients and is not intended for statistical inference. The left side horizontal bars indicate the total number of candidates per patient (Set Size). Patients 1–7 are ordered by set size (from lowest to highest). The black dots represent the number of unique miRNAs for patients. The black dots connected with lines represent patient miRNAs intersections. The vertical bars show the number (intersection size) and the name of the miRNAs in each overlap.

## 4 Discussion

To support conventional imaging techniques such as MRI for detecting tumor recurrence in GBM and to avoid unnecessary surgical procedures, we aimed to investigate a complementary liquid biopsy approach using whole-blood samples (3, 24). This method eliminates the labor-intensive isolation of microvesicles, allowing a minimally invasive approach (14, 25).

In this pilot “proof-of-concept” study, we aimed to validate miRNA recurrence-associated biomarkers by qRT-PCR that were previously identified during the NGS screening phase (18). The candidate miRNAs were selected based on a predefined three-stage filtering strategy to identify biologically significant miRNA candidates associated with GBM recurrence. The first filter considered miRNAs expressed in both primary tumor tissues and blood samples prior to surgery. The second filter selected miRNAs showing at least a two-fold decrease in expression after surgery. Based on the assumption that surgical resection significantly reduces or removes the tumor mass, the expression levels of tumor-associated miRNAs in peripheral blood are expected to decrease accordingly. Finally, the third filter identified miRNAs that showed a fold-change increase to pre-surgery levels or even exceeded them at the time of radiologically confirmed recurrence (18). This pattern was designed to identify tumor-associated miRNAs whose levels decrease after tumor resection and reappear during disease progression. A total of 45 miRNAs identified by NGS were selected among seven patients, and *miR-21* was also included based on its previously reported significance as a promising GBM-associated miRNA biomarker (22, 26–28). We performed a methodological validation of the identified miRNA candidates by NGS employing TaqMan chemistry qRT-PCR to ensure the highest standards in the use of this gold-standard technique for gene expression analysis and to ensure reproducibility across platforms. Nevertheless, an independent validation on a different cohort is still required as the prerequisite for further studies employing the diagnostic significance of our findings. It is important to note that all 46 miRNAs were analyzed in all patients, regardless of whether they had been identified as patient-specific NGS candidates. Thus, the qRT-PCR validation served not only as a technical confirmation of the candidates identified by NGS but also allowed for the search for candidates that were not selected as part of the patient-specific NGS filtering process. This enabled the detection of additional miRNAs that were not identified in individual patients by NGS, but nevertheless exhibited the predefined postoperative expression pattern when analyzed by qRT-PCR. This comprehensive qRT-PCR validation is relevant because some miRNAs can be overlooked by NGS analysis due to differences in detection sensitivity, read depth, pre-processing, filter thresholds, or platform-specific technical variations (29, 30).

A major challenge in this study was selecting an appropriate normalization strategy for qRT-PCR analysis. The normalization of miRNAs remains a contentious issue, particularly in studies of biomarkers from body fluids, where it is difficult to identify generally stable endogenous reference genes (31). In the present study, three commonly used reference targets were evaluated: *hsa-miR-103a-3p*, which has been described in the context of GBM analysis*; hsa-miR-16-5p*, which is recommended by Thermo Fisher for TaqMan miRNA assay cards; and *18S rRNA* as a widely used reference RNA. In addition, median, median ΔCt, and unnormalized raw Ct values were assessed.

The choice of normalization strategy significantly affected the calculated GE patterns. Therefore, a so called “plausibility analysis” was introduced to identify any biases caused by normalization. This approach investigated whether the normalized Ct values showed a systematic shift relative to the pre-surgery sample for each patient. A comprehensive shift affecting ≥70% of the analyzed miRNAs was considered implausible, as, according to the patient-specific NGS screening results, differential expression was expected only in a subset of the miRNAs examined. In other words, the majority of miRNAs should remain relatively unaltered over time, and a general upward or downward shift would suggest technical bias rather than genuine biological regulation. Using this approach, normalization with individual reference genes, including *hsa-miR-103a-3p*, *hsa-miR-16-5p*, and *18S rRNA*, was more prone to systematic bias (**Figure 2**, **Table 2**). This finding supports the known difficulty of identifying a single housekeeping miRNA or RNA species that remains stable across different patients, sampling time points, disease states, and treatment phases (32). In contrast, median ΔCt normalization and unnormalized raw Ct values showed the most robust and least biased performance in this dataset. This is consistent with previous observations suggesting that mean-based normalization approaches may outperform single miRNA reference genes in qRT-PCR-based miRNA studies (23).

Using the two most plausible approaches, namely unnormalized raw Ct values and median ΔCt normalization, the agreement between NGS miRNAs and qRT-PCR validation ranged from approximately 24% to 28% (**Table 3**). Although this overlap appears limited, incomplete concordance between NGS and qRT-PCR is expected and has been reported previously (23). Several factors may explain this discrepancy. NGS and qRT-PCR differ in their technical principles, dynamic ranges, sensitivity, normalization requirements, and data-processing pipelines. In addition, the present study did not evaluate a simple two-group comparison, but rather a complex GE pattern across several clinical time points. This makes validation more challenging, as a miRNA must follow the expected post-surgery decrease and recurrence-associated increase over the follow-up period. In addition, qRT-PCR analysis was performed using the same RNA samples that had already been used for NGS screening (dependent validation). Consequently, this study represents an analytical and methodological validation rather than an independent clinical validation. Whilst this approach is valuable for assessing platform concordance and the robustness of the miRNA candidates, future studies should validate these findings in an independent GBM cohort with a larger number of patients and prospectively collected samples.

Interestingly, qRT-PCR revealed greater overlap between patients than the NGS screening results (**Figure 4**). Whilst NGS largely revealed patient-specific miRNA profiles with small overlap between patients, the more comprehensive qRT-PCR validation identified several miRNAs that exhibited the expected expression patterns across multiple patients. The most common candidates were *hsa-miR-424-3p* and *hsa-miR-362-5p*, both of which were present in 5 of the 7 patients. This finding suggests that some recurrent miRNA signals may have been overlooked in NGS candidate selection, possibly due to patient-specific filtering, low frequency, or technical limitations of the sequencing. Among the identified candidates, *hsa-miR-424-3p* is of particular interest because it has been associated with tumor-related processes in several cancer types (33). Li et al. reported an association between *hsa-miR-424-3p*, chemoresistance, and tumor progression in gastric cancer. Although these findings were not obtained in GBM, they support a potential role for *miR-424-3p* in cancer biology (34). Furthermore, *hsa-miR-362-5p* has previously been described, in combination with *miR-6721-5p*, as a biomarker candidate for distinguishing grade 4 GBM from lower-grade gliomas (35). *Hsa-miR-22-5p*, which was shared by four patients, is also of biological interest. Han et al. reported that the lncRNA *MIR22HG* could promote GBM progression through the formation of *miR-22-5p* and *miR-22-3p*, as well as through the involvement of the Wnt/β-catenin signaling pathway (36). These findings suggest that the overlapped miRNAs may be promising candidates for further investigation. However, their biological relevance to GBM recurrence is still unclear. This study focused on longitudinal expression patterns rather than mechanistic function. Therefore, further experimental studies are required to determine whether these miRNAs contribute directly to tumor progression, response to treatment, or the biology of recurrence, or whether they reflect secondary tumor-associated processes. In particular, *miR-21*, which was included in the study due to its frequent mention in the GBM literature, did not exhibit the predefined expression pattern in any patient (22, 26, 27). This does not necessarily contradict earlier reports that describe *miR-21* as a GBM-associated biomarker. Rather, this suggests that a miRNA known to be associated with tumor presence or malignancy does not necessarily exhibit the specific post-surgery decline and the increase associated with recurrence required in this study. Thus, the present analysis underscores the importance of distinguishing between general disease-associated biomarkers and longitudinal monitoring biomarkers suitable for follow-up.

There are several limitations to consider in our study. First, the cohort size is small with only seven patients, which reflects the exploratory nature of this proof-of-concept study and limits the statistical power and applicability of the results. Second, the longitudinal design required multiple time points per patient; however, variability in timing and frequency of blood sampling certainly impacted the GE analysis. Third, whilst the plausibility-based normalization assessment helped to identify biased normalization strategies, there is currently no universally accepted gold standard for the normalization of circulating miRNAs. Finally, because all enrolled patients in our proof-of-concept study developed tumor recurrence, diagnostic accuracy metrics such as sensitivity, specificity, and the ROC curve could not be calculated; consequently, independent validation is still required.

In summary, this study demonstrates the potential of whole-blood-based liquid biopsy as a minimally invasive approach for monitoring GBM recurrence during clinical follow-up. Although the agreement between NGS and qRT-PCR was moderate, qRT-PCR validation identified additional miRNAs with the expected longitudinal expression pattern, including several candidates present in multiple patients. In particular, *hsa-miR-424-3p*, *hsa-miR-362-5p*, and *hsa-miR-22-5p* appear to be promising candidates for further validation in larger independent GBM cohorts. These findings suggest that profiling circulating miRNAs could complement conventional imaging by providing molecular information about tumor dynamics over time and allow for a more comprehensive, individualized aftercare. However, these results should be considered preliminary. Larger prospective studies with standardized sampling protocols, independent patient cohorts, and close correlation with radiological and clinical findings are needed to validate the clinical utility of this simplified liquid biopsy approach and to determine whether circulating miRNA signatures can reliably support the early detection of GBM recurrence.

## Data Availability

Original datasets are available in a publicly accessible repository: The original contributions prese nted in the study are publicly available. This data can be found here: Muhtadi, Razan (2026). TaqMan Assay Cards (qRT-PCR) Raw Data. figshare. Dataset. https://doi.org/10.6084/m9.figshare.33942520.

## Supplementary material

The Supplementary Material for this article can be found online at: https://www.frontiersin.org/articles/10.3389/fonc.2026.1938611/full#supplementary-material

Supplementary TABLE 1 This table shows the 46 genes included in the validation step, the two reference genes (hsa-miR-103a-3p and hsa-miR-16-5p), and their gene symbols, assay IDs, and functions.
